# General practitioner advice on physical activity: Analyses in a cohort of older primary health care patients (getABI)

**DOI:** 10.1186/1471-2296-12-26

**Published:** 2011-05-10

**Authors:** Timo Hinrichs, Anna Moschny, Renate Klaaßen-Mielke, Ulrike Trampisch, Ulrich Thiem, Petra Platen

**Affiliations:** 1 Department of Sports Medicine and Sports Nutrition, Ruhr-University Bochum, 44780 Bochum, Germany; 2 Department of Medical Informatics, Biometry and Epidemiology, Ruhr-University Bochum, 44780 Bochum, Germany; 3 Department of Geriatrics, Ruhr-University Bochum, Marienhospital Herne, 44627 Herne, Germany

**Keywords:** aged, physical activity, family physicians, primary health care, chronic disease, public health

## Abstract

**Background:**

Although the benefits of physical activity for health and functioning are recognized to extend throughout life, the physical activity level of most older people is insufficient with respect to current guidelines. The primary health care setting may offer an opportunity to influence and to support older people to become physically active on a regular basis. Currently, there is a lack of data concerning general practitioner (GP) advice on physical activity in Germany. Therefore, the aim of this study was to evaluate the rate and characteristics of older patients receiving advice on physical activity from their GP.

**Methods:**

This is a cross-sectional study using data collected at 7 years of follow-up of a prospective cohort study (German epidemiological trial on ankle brachial index, getABI). 6,880 unselected patients aged 65 years and above in the primary health care setting in Germany were followed up since October 2001. During the 7-year follow-up telephone interview, 1,937 patients were asked whether their GP had advised them to get regular physical activity within the preceding 12 months. The interview also included questions on socio-demographic and lifestyle variables, medical conditions, and physical activity. Logistic regression analysis (unadjusted and adjusted for all covariables) was used to examine factors associated with receiving advice. Analyses comprised only complete cases with regard to the analysed variables. Results are expressed as odds ratios (ORs) with 95% confidence intervals (95% CI).

**Results:**

Of the 1,627 analysed patients (median age 77; range 72-93 years; 52.5% women), 534 (32.8%) stated that they had been advised to get regular physical activity. In the adjusted model, those more likely to receive GP advice on physical activity were men (OR [95% CI] 1.34 [1.06-1.70]), patients suffering from pain (1.43 [1.13-1.81]), coronary heart disease and/or myocardial infarction (1.56 [1.21-2.01]), diabetes mellitus (1.79 [1.39-2.30]) or arthritis (1.37 [1.08-1.73]), and patients taking a high (> 5) number of medications (1.41 [1.11-1.80]).

**Conclusions:**

The study revealed a relatively low rate of older primary health care patients receiving GP advice on physical activity. GPs appeared to focus their advice on patients with chronic medical conditions. However, there are likely to be many more patients who would benefit from advice.

## Background

A high percentage of older adults (e.g., around 80% of the over 80-year-olds in Germany [1]) suffer from at least two chronic medical conditions. Regular physical activity is beneficial not only for the prevention but also for the management of many chronic conditions (e.g., coronary heart disease, diabetes, osteoporosis) [2,3]. Research suggests that even highly aged and frail persons may profit from physical activity with regard to physical functioning, mobility, and health-related quality of life [4-6]. Compared to current recommendations, the physical activity level of most older adults is insufficient [3,7]. Chronically ill persons are even less active than their healthy counterparts [8,9]. Thus, the promotion of physical activity among older adults should be a major public health concern. A greater understanding of opportunities for increasing the physical activity level of the elderly population is necessary.

The primary health care setting may offer a chance to influence older adults' physical activity behaviour [10]. The general practitioner (GP) is able to reach a large proportion of the elderly population, as a high percentage of elderly people regularly consult a GP for health problems [11,12]. The offer of a regular contact point for all sorts of health problems creates positive interaction, improves communication, and fosters the development of a relationship of trust between patients and their GPs [13-15]. This trusting relationship is essential for patient compliance [16]. Schofield et al. [17] report that the GP is the most trusted source of physical activity information, especially among older adults and those with multiple chronic diseases.

The management of physical inactivity in general practice is a complex process that usually starts with the assessment of physical activity [18]. Within all steps of the management process, the GP has to take the patient's individual health problems as well as environmental and personal factors into account [19]. This is time consuming and reduces the feasibility of managing physical inactivity in general practice [18]. Even though a number of physical activity counselling programmes administered through primary health care have been shown to be feasible and cost-effective strategies for promoting physical activity [20-25], systematic reviews performed by the US Preventive Services Task Force [26] and by Hudon et al. [27] concluded that there is conflicting evidence regarding the effectiveness of physician counselling on physical activity. Nevertheless, all of these authors seem to believe in the concept and recommend integrating further personnel (e.g. practice nurses) into the management process. The wide acceptance of the concept is supported by the fact that a number of professional organizations (including the American College of Preventive Medicine, the American Heart Association [AHA] and the American College of Sports Medicine [ACSM]) state that physical activity advice should be incorporated into routine patient visits in primary health care as a first step towards raising the physical activity level of patients [3,28]. In 2007, ACSM and AHA even launched the global-wide initiative "Exercise is Medicine™" that calls on physicians to prescribe exercise to their patients [29].

Currently, there is a lack of data concerning GP advice on physical activity in German primary health care. The aim of this study was to evaluate the rate and the characteristics of elderly patients receiving physical activity advice from their GP based upon patients' self-reports.

## Methods

### Design and participants

The "German Epidemiological Trial on Ankle Brachial Index" (getABI) is a prospective cohort study. Its design and methods have been described elsewhere in greater detail [30,31]. In short, 344 GP practices across Germany supervised by 34 vascular physicians in their vicinity took part in the study. An assessment of primary health care attendees, irrespective of their reason for seeing the GP, was conducted in a predetermined week in October 2001. Each GP consecutively recruited on average 20 (maximum 25) eligible patients fulfilling the inclusion criteria (age ≥ 65 years, being legally competent and able to cooperate appropriately, and providing written informed consent). The only exclusion criterion was life expectancy ≤ 6 months. Ultimately, a total of 6,880 primary health care patients were included in the cohort. Within the 7-year follow-up period, 1,302 patients died. The remaining 5,578 patients were contacted by letter and by one telephone call to evaluate their willingness to participate in the computer-assisted telephone interview at the 7-year follow-up. 196 patients were unable to participate in the interview; another 3,445 patients did not participate in the interview for several other reasons (not reachable, did not want to be contacted by telephone, refused to participate in the telephone interview). Finally, a sample of 1,937 patients (response rate 34.7%) took part in the telephone interviews at the 7-year follow-up. Comparing these participants to non-participants revealed the following significant differences: participants were younger at baseline (median age (range): 70 (65-85) years vs. 72 (65-91) years), were more often male (46.7% vs. 35.7%), and were better educated (qualification higher than basic secondary school: 40.2% vs. 27.1%). Results reported in this paper mainly refer to cross-sectional data collected during the 7-year follow-up interviews.

The study was approved by the ethics committees of Heidelberg University and the Ruhr-University Bochum (Germany) and was conducted according to the "Good Epidemiological Practice" recommendations issued by the "German Working Group Epidemiology" [32].

### Outcome

#### GP advice on physical activity

Participants were asked if their GP had advised them to get regular physical activity within the preceding 12 months. The response options were: "Yes, she/he advised me to be regularly physically active"/"No, she/he didn't give me any advice on physical activity"/"No, she/he advised me to rest". Participants who stated that their GP had advised them to rest were excluded from further analyses. The reason for this advice could not be evaluated, but medical reasons justifying this recommendation appeared likely.

### Covariables

#### Socio-demographic variables

At baseline, the GP documented the participants' sex, date of birth, and education level (no qualification/completed basic secondary school/vocational school/university entrance qualification). The place of birth was elicited by telephone interview at 5-year follow-up.

#### Number of GP visits

The number of GP visits during the preceding 3 months was assessed by telephone interview (7-year follow-up).

#### Walking ability and falls

Current walking ability (no walking aid/cane/rollator/wheelchair-bound/bed-ridden) and history of falls (no/yes) within the past 12 months were assessed by telephone interview (7-year follow-up). Falls were defined as "an unexpected event in which the participants come to rest on the ground, floor, or lower level" [33].

#### Pain

Pain (no/yes) within the previous 3 months (permanent and/or intermittent) was assessed by telephone interview (7-year follow-up).

#### Cardiovascular risk factors

The current smoking status (no/yes) was documented at baseline. The participants' waist circumference was measured by study personnel in the GP's practice by standard protocol at the 5- and the 7-year follow-up. Waist circumference at 7-year follow-up was used for analysis. If this value was absent, waist circumference at 5-year follow-up was used.

#### Medical conditions and number of medications

The presence of chronic diseases and the number of medications were assessed by telephone interview at 7-year follow-up. Participants were asked whether a physician had ever diagnosed one of the following chronic diseases (no/yes): arterial hypertension, coronary heart disease (CHD), myocardial infarction, chronic heart failure (CHF), diabetes mellitus, peripheral arterial disease (PAD), chronic obstructive pulmonary disease (COPD), arthritis (degenerative or rheumatoid), osteoporosis.

#### Sporting activities

Performance and duration of sporting activities (cycling, exercising and/or strength training, organized sports groups, other sporting or leisure activities that caused sweating) during the preceding week were assessed during the telephone interview (7-year follow-up) using the PRISCUS Physical Activity Questionnaire [34].

### Statistical analysis

Descriptive statistics are presented as counts and percentages. A logistic regression analysis (both unadjusted and adjusted for all covariables) was performed to assess the odds of receiving advice to get regular physical activity (no/yes). The following 20 binary covariables were included in the statistical model (each no/yes, if not indicated otherwise): sex (female/male), age (</≥ 80 years), place of birth (outside/in Germany), qualification (no graduation or completed basic secondary school/higher than basic secondary school qualification), number of GP visits during the preceding 3 months (0-3/> 3 visits), need for a walking aid, falls within the preceding 12 months, pain during the preceding 3 months, currently smoking at baseline, waist circumference (women </≥ 88 cm; men </≥ 102 cm) [35], arterial hypertension, CHD and/or myocardial infarction, CHF, diabetes mellitus, PAD, COPD, arthritis, osteoporosis, number of medications (unmedicated or ≤/> 5), sporting activities (</≥ 2.5 hours per week). Participants with incomplete values in the outcome or the covariables were excluded from analyses. The 95% confidence interval was calculated for every odds ratio.

The recruitment method for the getABI cohort resulted in clustering of patients within GPs. Because advice within the same GP is likely to be correlated, adjustment at GP-level would be preferable. As this was not feasible because of the large number of GPs participating in the study, an additional sensitivity analysis (logistic regression with an additional centre variable) was performed. The centre variable was defined as the percentage of patients receiving advice from the respective GP.

## Results

### Participant characteristics

Of the 1,937 participants (median age 77; range 72-93 years; 53.3% women) in the 7-year follow-up telephone interviews, 310 participants were excluded either because of incomplete data (n = 266) or because they stated that their GP had advised them to rest (n = 54). Comparing these cases to those analysed revealed differences in the following parameters (excluded vs. analysed): number of GP visits (> 3 in past 3 months: 29.9% vs. 19.7%), need for a walking aid (yes: 24.0% vs. 15.6%), and number of medications (> 5: 63.1% vs. 52.0%). The two groups did not substantially differ regarding sex and age, cardiovascular risk factors, various chronic medical conditions, falls and pain, or sporting activity level.

A total of 1,627 participants (median age 77 years; range 72-93 years; 52.5% women) were ultimately included in the analyses. Table 1 shows the characteristics of these participants, 534 (32.8%) of whom stated that their GP had advised them to get regular physical activity within the preceding 12 months.

**Table 1:** Characteristics of participants included in (n = 1,627) and participants excluded (n = 310) from the analyses

	included		excluded	
	
	n	%	n	%
**Outcome**				
Receiving general practitioner advice on physical activity	534	32.8	76	32.8

**Socio-demographic variables**				
Male sex	773	47.5	131	42.3
Age ≥ 80 years	545	33.5	118	38.1
Place of birth Germany	1474	90.6	291	93.9
Education level higher than basic secondary school qualification	670	41.2	108	37.8

**Cardiovascular risk factors**				
Currently smoking (baseline)	110	6.8	17	5.5
Waist circumference^a^: women ≥ 88 cm; men ≥ 102 cm	1024	62.9	170	66.9

**Medical conditions and medication**				
Hypertension	1046	64.3	201	65.3
Coronary heart disease and/or myocardial infarction	408	25.1	90	29.2
Chronic heart failure	303	18.6	67	21.8
Diabetes mellitus	406	25.0	73	23.7
Peripheral arterial disease	190	11.7	39	12.7
Chronic obstructive pulmonary disease	185	11.4	53	17.2
Arthritis (degenerative or rheumatoid)	568	34.9	120	39.0
Osteoporosis	209	12.8	65	21.1
> 5 medications	846	52.0	195	63.1

**> 3 GP visits (preceding 3 months)**	321	19.7	90	29.9

**Need for walking aid**	254	15.6	73	24.0

**Falls (past 12 months)**	347	21.3	81	26.3

**Pain (past 3 months)**	864	53.1	167	54.0

**Sporting activities < 2.5 hours per week**	1036	63.7	103	70.5

^a ^Waist circumference at 7-year follow-up was used for analysis. If this value was missing, waist circumference at 5-year follow-up was used.

### Odds ratios for receiving GP advice on physical activity

The results of the logistic regression analysis (both unadjusted and adjusted for all covariables) are shown in Table 2.

**Table 2:** Association between patient characteristics and receiving general practitioner advice on physical activity (logistic regression model; n = 1,627)

	% receiving advice	Crude odds ratio [95% confidence interval]	**Adjusted odds ratio^b^** [95% confidence interval]
**Socio-demographic variables**			
Sex			
Female	30.0	1	1
Male	36.0	1.31 [1.07;1.61]*	1.34 [1.06;1.70]*
Age			
< 80 years	34.6	1	1
≥ 80 years	29.4	0.79 [0.63;0.98]*	0.78 [0.61;1.00]
Birthplace			
Outside Germany	39.2	1	1
Germany	32.2	0.74 [0.52;1.04]	0.81 [0.57;1.17]
Education level			
No qualification or basic secondary school qualification	32.4	1	1
Higher than basic secondary school qualification	33.4	1.05 [0.85;1.29]	1.11 [0.89;1.39]

**Cardio-vascular risk factors**			
Currently smoking (baseline)			
No	33.0	1	1
Yes	30.9	0.91 [0.60;1.38]	0.92 [0.59;1.43]
Waist circumference^a^			
Women < 88 cm; men < 102 cm	28.9	1	1
Women ≥ 88 cm; men ≥ 102 cm	35.2	1.34 [1.08;1.66]*	1.19 [0.94;1.51]

**Medical conditions and medications**			
Hypertension			
No	28.6	1	1
Yes	35.2	1.36 [1.09;1.69]*	1.09 [0.86;1.38]
Coronary heart disease and/or myocardial infarction			
No	29.0	1	1
Yes	44.1	1.93 [1.53;2.43]*	1.56 [1.21;2.01]*
Chronic heart failure			
No	31.6	1	1
Yes	38.0	1.32 [1.02;1.71]*	1.02 [0.77;1.35]
Diabetes mellitus			
No	28.6	1	1
Yes	45.6	2.09 [1.66;2.64]*	1.79 [1.39;2.30]*
Peripheral arterial disease			
No	31.3	1	1
Yes	44.2	1.74 [1.28;2.36]*	1.31 [0.94;1.83]
Chronic obstructive pulmonary disease			
No	32.0	1	1
Yes	39.5	1.39 [1.01;1.90]*	1.23 [0.88;1.72]
Arthritis (degenerative or rheumatoid)			
No	29.0	1	1
Yes	40.0	1.63 [1.32;2.02]*	1.37 [1.08;1.73]*
Osteoporosis			
No	32.4	1	1
Yes	35.9	1.17 [0.86;1.58]	1.07 [0.77;1.50]
Number of medications			
unmedicated or ≤ 5	24.8	1	1
> 5	40.2	2.03 [1.64;2.52]*	1.41 [1.11;1.80]*

**Number of general practitioner visits (preceding 3 months)**			
0-3 visits	31.0	1	1
> 3 visits	40.2	1.50 [1.16;1.92]*	1.28 [0.97;1.67]

**Need for walking aid**			
No	32.2	1	1
Yes	36.2	1.20 [0.90;1.59]	0.83 [0.61;1.15]

**Falls (preceding 12 months)**			
No	32.6	1	1
Yes	33.7	1.05 [0.82;1.35]	1.04 [0.80;1.36]

**Pain (preceding 3 months)**			
No	26.9	1	1
Yes	38.1	1.67 [1.36;2.07]*	1.43 [1.13;1.81]*

**Sporting activities**			
< 2.5 hours per week	33.0	1	1
≥ 2.5 hours per week	32.7	0.99 [0.80;1.23]	0.96 [0.76;1.22]

* 95% Confidence interval not including 1

^a ^Waist circumference at 7-year follow-up was used for analysis. If this value was missing, waist circumference at 5-year follow-up was used.

^b ^Adjusted for all other variables in the table

The unadjusted regression analysis revealed that the odds of receiving GP advice on physical activity were about one-third higher for male patients. This value remained stable after adjustment for all covariables. The odds of receiving advice were about 20% lower for older patients (≥ 80 years) than for younger patients (< 80 years). This percentage was also unaffected by adjustment.

Participants taking a high (> 5) number of medications and those suffering from pain were more likely to receive GP advice on physical activity (unadjusted and adjusted model). All chronic conditions except osteoporosis showed a positive association with GP advice on physical activity in the unadjusted analysis. The variables diabetes mellitus and CHD and/or myocardial infarction accounted for the highest odds ratios (2.09 and 1.93) for receiving advice. As expected, due to the known interdependencies between many chronic conditions, the number of medications and pain, the odds ratios for all these variables decreased with adjustment.

Factors not found to be associated with receiving GP advice on physical activity, either in the unadjusted or in the adjusted model were: migration background, education level, need for a walking aid, history of falls, smoking status, osteoporosis, and performance of sporting activities.

The sensitivity analysis with an additional centre variable led to only marginal changes of the shown results. The direction of effects remained the same.

## Discussion

The present study investigated the rate and the factors associated with GP advice on physical activity as reported by study participants. To the authors' knowledge, no other research has reported physical activity advice rates in Germany to date.

Only about one-third of elderly primary health care patients stated that their GP had given them advice to get regular physical activity within the preceding year. While this rate is much lower than desirable, it is in line with findings of previous studies performed in the US and in Australia that generally report counselling rates of lower than 40% [36-41].

Most previous studies included adults of all ages [36-39]. As an example, Glasgow et al. [36] reported that 28% of respondents from a diverse sample of US adults reported receiving physician advice to increase their physical activity level. Wee et al. [37] found that 34% of all patients who had seen a physician for a medical check-up in the preceding year had received counselling to begin or to continue to exercise. An Australian survey [39] revealed a rate of 24% of adults receiving GP advice on physical activity. Studies focusing on the elderly population are rare. A US study [40] in a sample of 893 older adults aged 64 to 95 years found that 24% received a physician's recommendation to exercise within the preceding year. Another US study [41] in a sample of 141 elderly managed care patients found that receiving health care provider advice to be more physically active was associated with the interest in participating in a future interventional physical activity study. Advice on physical activity had been given to 55% of the interested participants, whereas only 20% of the uninterested participants had ever been advised. Only Balde et al. [42], who investigated a homogeneous sample of low-income senior citizens living in public housing, found a considerably higher counselling rate than the other authors, namely 62%.

There may be a wide range of reasons for rather low counselling rates across the studies. A number of barriers preventing GPs from offering counselling about physical activity have been reported: competing demands of providing a broad range of preventive and non-preventive services, time constraints during consultations, lack of educational resources and of formal clinician training in physical activity counselling, preference of patients for drug treatment, and lack of reimbursement [39,42-45]. Dupen et al. [46] noted that physical activity is under-recognized by medical journals and magazines frequently read by Australian GPs in comparison to other traditional cardiovascular disease risk factors (e.g., arterial hypertension or hypercholesterolemia), which could be another explanation for their counselling behaviour. The literature shows that the above-mentioned barriers are not unique to physical activity counselling, but also apply to counselling for other complex behaviours like nutrition [47-49], where counselling rates are similarly low [50,51]. These findings suggest that future counselling programmes for the primary health care setting will have to find ways to reduce the burden on GPs.

The differences in advice rates in association to patient characteristics suggest that GP counselling behaviour is influenced by those characteristics. Our study suggests that older patients (≥ 80 years) may be less likely to receive physical activity advice than younger patients (< 80 years). This finding is supported by Schonberg et al. [52], who reported that women aged ≥ 75 years were considerably less likely to receive physical activity advice from their physician than women aged 50 to 64 years. GPs may either not be aware that physical activity can be beneficial even for highly aged individuals, or they may have concerns with regard to negative health outcomes or be uncertain as to the optimal mode, frequency, amount and intensity of exercise for this age group [53,54].

The present study found that men were more likely to receive GP advice then women. This is in line with results from previous studies in adults of all ages [39,45] and from a study in older adults [42]. This could be a sign of the failure to recognize some health issues in women by GPs, as has already been acknowledged for issues such as cardiovascular disease [55,56]. However, the reasons for this finding are unclear.

The present study revealed that the presence of diabetes mellitus, CHD and/or myocardial infarction, arthritis, a high (> 5) number of medications, and pain were independently associated with receiving GP advice on physical activity. In their survey among adults of all ages, Wee et al. [37] found that physical activity advice was more likely to be given to patients with cardiac disease or diabetes mellitus. Kreuter et al. [57] reported that patients with diabetes mellitus or high blood pressure were more likely to receive advice. Glasgow et al. [36] as well as Eakin et al. [39] observed that rates of physician advice increased with the presence of chronic conditions (without specifying the conditions). Furthermore, previous studies in adults of all ages [37,39] and in older adults [40,42] found that overweight or obese participants were more likely to receive advice on physical activity. In our study, waist circumference was not independently associated with GP advice after multivariate adjustment. Bull et al. [45] performed a survey among GPs concerning their behaviour regarding promotion of physical activity. Their results confirm the findings from surveys among patients: GPs indicated that they most frequently recommend physical activity to "patients with conditions that could benefit from physical activity" and to "patients in need of weight management". In sum, research suggests that GPs tend to counsel patients whose health is already compromised and who they judge as likely to benefit from physical activity. Especially patients with heart disease, diabetes mellitus or overweight seem to profit from GPs' counselling behaviour. Unfortunately, GPs seem to neglect the potential benefit of physical activity in the following scenarios: 1. for the prevention of diseases, and 2. as a therapeutic measure in other medical conditions (e.g. osteoporosis, history of falls).

One limitation of the present study is the fact that data are based on participant self-report. Self-reported chronic conditions were not double-checked with GP documentation. The recall bias may underestimate actual advice rates. However, participants' recall may also be regarded as a true reflection of the sustainability of the GP's advice. The present study did not evaluate GP characteristics (e.g. their own attitude towards physical activity) that may be associated with advice on physical activity.

In addition, some data used for analysis may have changed within the follow-up period. This limitation refers to the variables smoking status (baseline data) and waist circumference (7-year follow-up or 5-year follow-up data).

Another limitation of this study is clustering of patients within GPs due to the recruitment method for the getABI cohort. Advice within the same GP is likely to be correlated. Therefore, confidence intervals in our model might be slightly too small. A sensitivity analysis (logistic regression with an additional centre variable) led to only marginal changes of our findings. This indicates that there seems to be only a small correlation of the centre variable with the other explanatory variables. However, this additional variable may not fully explain the centre effect. Thus we cannot exclude a certain degree of underestimation, but the effect should be minor.

The response rate in the 7-year follow-up telephone interview was 34.7%. As expected, participants were younger and better educated compared to non-participants. Furthermore, it can be assumed that the willingness and ability to continue to participate in a longitudinal trial after 7 years is higher in healthier persons. Participants who had moved to a nursing home during the follow-up period were no longer able to participate. Therefore, there is most likely a selection towards the fitter patients from baseline to the 7-year follow-up in the getABI cohort. Nevertheless, some of the selection bias would have occurred anyway, even if a completely new cross-sectional study would have been set up. The interest in participating in such a trial is always lower in persons with disabilities or multiple morbidities. Therefore, our results should be regarded as relevant for a population of relatively fit seniors who are still able to visit their GP and take part in a telephone interview.

## Conclusions

This exploratory study revealed a relatively low rate of GP advice on physical activity among elderly primary care patients in Germany. GPs appeared to focus their physical activity advice on patients with selected medical conditions, even though many more patients would be likely to benefit from such advice. A deeper understanding of GPs' counselling behaviour is needed to develop effective counselling programmes for the primary health care setting.

## Competing interests

The authors declare that they have no competing interests.

## Authors' contributions

TH and PP obtained the research grant for the project "Physical activity, multimorbidity and polypharmacy in the elderly" within the PRISCUS research cooperation and initiated the specific data collection in the getABI cohort. TH and AM conceived the research question, edited the data, performed the statistical analyses, interpreted the data, and drafted the manuscript. RKM participated in data preparation and double-checked the statistical analyses. UT developed the PRISCUS Physical Activity Questionnaire and participated in the interpretation of data. UTh is coordinator of the PRISCUS research cooperation, including the supervision of data collection in the getABI cohort; he also participated in the interpretation of data. All authors revised the manuscript critically for important intellectual content. All authors approved the version to be published.

## Pre-publication history

The pre-publication history for this paper can be accessed here:

http://www.biomedcentral.com/1471-2296/12/26/prepub
